# (2*E*)-1-(4-Amino­phen­yl)-3-(2-thien­yl)prop-2-en-1-one ethanol hemisolvate

**DOI:** 10.1107/S1600536809037933

**Published:** 2009-09-26

**Authors:** Hoong-Kun Fun, Thawanrat Kobkeatthawin, Suchada Chantrapromma

**Affiliations:** a X-ray Crystallography Unit, School of Physics, Universiti Sains Malaysia, 11800 USM, Penang, Malaysia; b Department of Chemistry and Center of Excellence for Innovation in Chemistry, Faculty of Science, Prince of Songkla University, Hat-Yai, Songkhla 90112, Thailand; c Crystal Materials Research Unit, Department of Chemistry, Faculty of Science, Prince of Songkla University, Hat-Yai, Songkhla 90112, Thailand

## Abstract

In the title compound, C_13_H_11_NOS·0.5C_2_H_6_O, the chalcone derivative is close to planar, the dihedral angle between the thio­phene and 4-amino­phenyl rings being 3.1 (2)°. The thio­phene ring is disordered over two orientations with occupancies of 0.842 (3) and 0.158 (3). In the crystal structure, mol­ecules are linked into chains along the *b* axis by N—H⋯O hydrogen bonds. The chains are crosslinked *via* N—H⋯π inter­actions involving the thio­phene ring. The ethanol solvent mol­ecule is also disordered over two positions, each with an occupancy of 0.25.

## Related literature

For bond-length data, see: Allen *et al.* (1987 ▶). For related structures, see: Fun *et al.* (2009 ▶); Suwunwong *et al.* (2009 ▶). For background and applications of chalcones, see: Dimmock *et al.* (1999 ▶); Go *et al.* (2005 ▶); Ni *et al.* (2004 ▶); Patil & Dharmaprakash (2008 ▶). For the stability of the temperature controller used in the data collection, see: Cosier & Glazer (1986 ▶).
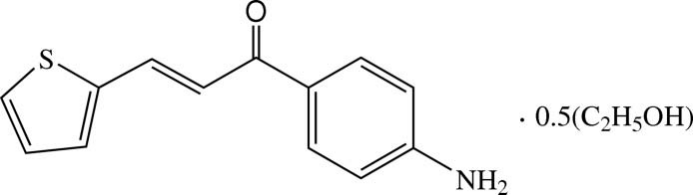


## Experimental

### 

#### Crystal data

C_13_H_11_NOS·0.5C_2_H_6_O
*M* *_r_* = 252.32
Orthorhombic, 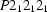

*a* = 5.1413 (1) Å
*b* = 13.9754 (2) Å
*c* = 18.2647 (2) Å
*V* = 1312.35 (3) Å^3^
*Z* = 4
Mo *K*α radiation
μ = 0.24 mm^−1^
*T* = 100 K
0.56 × 0.22 × 0.17 mm

#### Data collection

Bruker APEXII CCD area-detector diffractometer
Absorption correction: multi-scan (*SADABS*; Bruker, 2005 ▶) *T*_min_ = 0.879, *T*_max_ = 0.961
22258 measured reflections
4225 independent reflections
3893 reflections with *I* > 2σ(*I*)
*R*_int_ = 0.026

#### Refinement

*R*[*F* ^2^ > 2σ(*F* ^2^)] = 0.061
*wR*(*F* ^2^) = 0.205
*S* = 1.09
4225 reflections
188 parameters
14 restraints
H-atom parameters constrained
Δρ_max_ = 1.04 e Å^−3^
Δρ_min_ = −0.31 e Å^−3^
Absolute structure: Flack (1983 ▶), 1874 Friedel pairs
Flack parameter: 0.00 (12)

### 

Data collection: *APEX2* (Bruker, 2005 ▶); cell refinement: *SAINT* (Bruker, 2005 ▶); data reduction: *SAINT*; program(s) used to solve structure: *SHELXTL* (Sheldrick, 2008 ▶); program(s) used to refine structure: *SHELXTL*; molecular graphics: *SHELXTL*; software used to prepare material for publication: *SHELXTL* and *PLATON* (Spek, 2009 ▶).

## Supplementary Material

Crystal structure: contains datablocks global, I. DOI: 10.1107/S1600536809037933/ci2908sup1.cif

Structure factors: contains datablocks I. DOI: 10.1107/S1600536809037933/ci2908Isup2.hkl

Additional supplementary materials:  crystallographic information; 3D view; checkCIF report

## Figures and Tables

**Table 1:** Hydrogen-bond geometry (Å, °)

*D*—H⋯*A*	*D*—H	H⋯*A*	*D*⋯*A*	*D*—H⋯*A*
N1—H1*A*⋯O1^i^	0.86	2.16	2.931 (3)	149
N1—H1*B*⋯*Cg*1^ii^	0.86	2.80	3.597 (3)	156

Symmetry codes: (i) 
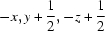
; (ii) 
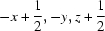
. *Cg*1 is the centroid of the S1/C10–C13 ring.

**Fig. 1.**
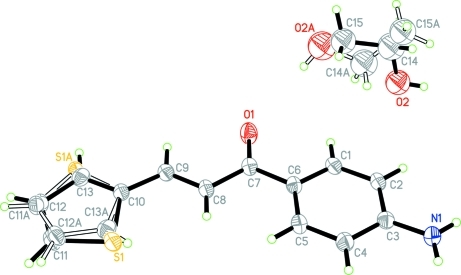
The molecular structure of the title compound, showing 50% probability displacement ellipsoids and the atom-numbering scheme. The minor disorder components are shown in open bonds.

**Fig. 2.**
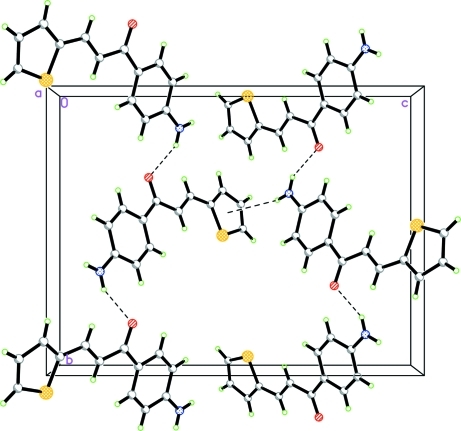
The crystal packing of the title compound, viewed along the a axis, showing chains running along the b axis. N—H···O hydrogen bonds and N—H···π interactions are shown as dashed lines. Only the major disorder component is shown. For clarity, the disordered ethanol solvent molecules are not shown.

## supplementary crystallographic information

### Comment

Chalcones have been reported to be responsible for a variety of biological activities such as analgesic, anti-inflammatory, antibacterial and antimycotic properties (Dimmock *et al.*, 1999; Go *et al.*, 2005; Ni *et al.*, 2004). Some of the synthetic chalcones have also been found to be non-linear optical (NLO) materials (Patil & Dharmaprakash, 2008). These interesting activities have led us to synthesize the title heteroaryl chalcone, (I), in order to study its NLO properties and biological activities. Herein we report the crystal strcuture of (I). The title compound crystallizes in orthorhombic noncentrosymmetric space group *P*2_1_2_1_2_1_ and therefore it is expected to exhibit second-order non-linear optic properties.

The molecule of the title heteroaryl chalcone (Fig. 1) exists in an *E* configuration with respect to the C8═C9 double bond [1.346 (3) Å], with C7—C8—C9—C10 torsion angle of 179.1 (2)°. The molecule is essentially planar as indicated by the dihedral angle between thiophene (C10–C13/S1) and 4-aminophenyl rings of 3.1 (2)°. Bond distances (Allen *et al.*, 1987) and angles show normal values and are comparable with those observed in closely related structures (Fun *et al.*,  2009; Suwunwong *et al.*, 2009).

In the crystal, molecules are linked into chains along the *b* axis through N—H···O hydrogen bonds (Fig. 2 and Table 1). The chains are interlinked via N—H···π interactions (Table 1) involving the C10-C13/S1 ring (Fig.2).

### Experimental

The title compound was synthesized by the condensation of 4-aminoacetophenone (0.40 g, 3 mmol) with thiophene-2-carboxaldehyde (0.28 ml, 3 mmol) in ethanol (30 ml) in the presence of 10% NaOH (aq) (5 ml). After stirring for 2 hr at room temperature, the resulting yellow solid was collected by filtration, washed with distilled water, dried and purified by repeated recrystallization from acetone. Yellow plate-shaped single crystals of the title compound suitable for *X*-ray structure determination were grown by slow evaporation of an ethanol solution at room temperature after several days, (m.p. 378–379 K).

### Refinement

The thiophene ring of the chalcone is disordered over two orientations with occupancies of 0.842 (3) and 0.158 (3). The same anisotropic displacement parameters were used for atoms pairs C12A/C11, C11A/C12 and C13A/C13. Atoms S1A, C11A, C12A, C13A and C10 were restrained to be coplanar. The ethanol solvent molecule is also disordered over two positions across a center of symmetry. Their occupanicies were initially refined to 0.248 (5) and 0.242 (5) and later both were fixed at 0.25. Both disorder components were refined isotropically. The C—O, C—C and O···C distances were restrained to 1.42 (1), 1.51 (1) and 2.43 (1) Å, respectively. All H atoms were placed in calculated positions, with N-H = 0.86 Å, C-H = 0.93-0.97 Å. The *U*_iso_ values were constrained to be 1.5*U*_eq_ of the carrier atom for methyl and hydroxyl H atoms and 1.2*U*_eq_(C) for the remaining H atoms. A rotating group model was used for the methyl groups. The highest residual electron density peak is located at 0.96 Å from H2B and the deepest hole is located at 0.30 Å from H14B. The final difference density features indicate that the solvent molecule may be disordered over multiple sites.

### Crystal data

**Table:**

C_13_H_11_NOS·0.5C_2_H_6_O	*D*_x_ = 1.277 Mg m^−^^3^
*M**_r_* = 252.32	Melting point = 378–379 K
Orthorhombic, *P*2_1_2_1_2_1_	Mo *K*α radiation, λ = 0.71073 Å
Hall symbol: P 2ac 2ab	Cell parameters from 4225 reflections
*a* = 5.1413 (1) Å	θ = 1.8–31.1°
*b* = 13.9754 (2) Å	µ = 0.24 mm^−^^1^
*c* = 18.2647 (2) Å	*T* = 100 K
*V* = 1312.35 (3) Å^3^	Plate, yellow
*Z* = 4	0.56 × 0.22 × 0.17 mm
*F*(000) = 532	

### Data collection

**Table:**

Bruker APEXII CCD area-detector diffractometer	4225 independent reflections
Radiation source: sealed tube	3893 reflections with *I* > 2σ(*I*)
graphite	*R*_int_ = 0.026
φ and ω scans	θ_max_ = 31.1°, θ_min_ = 1.8°
Absorption correction: multi-scan (*SADABS*; Bruker, 2005)	*h* = −7→7
*T*_min_ = 0.879, *T*_max_ = 0.961	*k* = −20→15
22258 measured reflections	*l* = −26→24

### Refinement

**Table:**

Refinement on *F*^2^	Secondary atom site location: difference Fourier map
Least-squares matrix: full	Hydrogen site location: inferred from neighbouring sites
*R*[*F*^2^ > 2σ(*F*^2^)] = 0.061	H-atom parameters constrained
*wR*(*F*^2^) = 0.205	*w* = 1/[σ^2^(*F*_o_^2^) + (0.1552*P*)^2^ + 0.2319*P*] where *P* = (*F*_o_^2^ + 2*F*_c_^2^)/3
*S* = 1.09	(Δ/σ)_max_ = 0.001
4225 reflections	Δρ_max_ = 1.04 e Å^−^^3^
188 parameters	Δρ_min_ = −0.31 e Å^−^^3^
14 restraints	Absolute structure: Flack (1983), 1874 Friedel pairs
Primary atom site location: structure-invariant direct methods	Flack parameter: 0.00 (12)

### Special details

**Table:**

Experimental. The crystal was placed in the cold stream of an Oxford Cryosystems Cobra open-flow nitrogen cryostat (Cosier & Glazer, 1986) operating at 120.0 (1) K.
Geometry. All esds (except the esd in the dihedral angle between two l.s. planes) are estimated using the full covariance matrix. The cell esds are taken into account individually in the estimation of esds in distances, angles and torsion angles; correlations between esds in cell parameters are only used when they are defined by crystal symmetry. An approximate (isotropic) treatment of cell esds is used for estimating esds involving l.s. planes.
Refinement. Refinement of *F*^2^ against ALL reflections. The weighted *R*-factor *wR* and goodness of fit *S* are based on *F*^2^, conventional *R*-factors *R* are based on *F*, with *F* set to zero for negative *F*^2^. The threshold expression of *F*^2^ > σ(*F*^2^) is used only for calculating *R*-factors(gt) *etc*. and is not relevant to the choice of reflections for refinement. *R*-factors based on *F*^2^ are statistically about twice as large as those based on *F*, and *R*- factors based on ALL data will be even larger.

### Fractional atomic coordinates and isotropic or equivalent isotropic displacement parameters (Å^2^)

**Table:**

	*x*	*y*	*z*	*U*_iso_*/*U*_eq_	Occ. (<1)
O1	0.4331 (4)	−0.19026 (12)	0.23694 (11)	0.0354 (4)	
N1	−0.3267 (4)	0.13702 (15)	0.34900 (11)	0.0303 (4)	
H1A	−0.3311	0.1971	0.3390	0.036*	
H1B	−0.4325	0.1135	0.3807	0.036*	
C1	0.0494 (4)	−0.07627 (15)	0.29945 (11)	0.0260 (4)	
H1	0.0568	−0.1408	0.3116	0.031*	
C2	−0.1363 (4)	−0.01898 (16)	0.33217 (11)	0.0257 (4)	
H2	−0.2512	−0.0453	0.3659	0.031*	
C3	−0.1526 (4)	0.07911 (15)	0.31464 (11)	0.0249 (4)	
C4	0.0225 (5)	0.11577 (16)	0.26252 (12)	0.0296 (4)	
H4	0.0135	0.1800	0.2495	0.035*	
C5	0.2078 (4)	0.05764 (16)	0.23027 (13)	0.0284 (4)	
H5	0.3214	0.0835	0.1960	0.034*	
C6	0.2273 (4)	−0.03956 (15)	0.24835 (10)	0.0232 (4)	
C7	0.4185 (4)	−0.10551 (15)	0.21554 (11)	0.0244 (4)	
C8	0.5911 (4)	−0.07082 (15)	0.15699 (12)	0.0256 (4)	
H8	0.5733	−0.0082	0.1405	0.031*	
C9	0.7742 (4)	−0.12715 (15)	0.12666 (11)	0.0251 (4)	
H9	0.7867	−0.1897	0.1436	0.030*	
C10	0.9527 (4)	−0.09779 (15)	0.06978 (11)	0.0241 (4)	
S1	0.95430 (16)	0.01724 (6)	0.03611 (4)	0.0312 (2)	0.842 (3)
C11	1.2101 (13)	−0.0077 (4)	−0.0214 (2)	0.0304 (8)	0.842 (3)
H11	1.2848	0.0368	−0.0529	0.037*	0.842 (3)
C12	1.2907 (12)	−0.1014 (4)	−0.0157 (4)	0.0293 (6)	0.842 (3)
H12	1.4268	−0.1277	−0.0425	0.035*	0.842 (3)
C13	1.1404 (12)	−0.1509 (4)	0.0372 (4)	0.0291 (11)	0.842 (3)
H13	1.1694	−0.2150	0.0481	0.035*	0.842 (3)
S1A	1.1592 (16)	−0.1790 (5)	0.0320 (5)	0.0257 (13)	0.158 (3)
C11A	1.312 (7)	−0.095 (2)	−0.020 (2)	0.0293 (6)	0.16
H11A	1.4432	−0.1078	−0.0536	0.035*	0.158 (3)
C12A	1.208 (7)	−0.006 (2)	−0.0088 (18)	0.0304 (8)	0.16
H12A	1.2513	0.0496	−0.0341	0.037*	0.158 (3)
C13A	1.005 (4)	−0.0026 (14)	0.0424 (12)	0.0291 (11)	0.16
H13A	0.9294	0.0517	0.0628	0.035*	0.158 (3)
O2	−0.2058 (19)	−0.2501 (8)	0.4350 (6)	0.060 (3)*	0.25
H2B	−0.3410	−0.2368	0.4565	0.089*	0.25
C14	0.013 (2)	−0.2424 (15)	0.4817 (8)	0.070 (4)*	0.25
H14A	0.0016	−0.2951	0.5161	0.084*	0.25
H14B	0.0012	−0.1832	0.5093	0.084*	0.25
C15	0.272 (2)	−0.2486 (12)	0.4437 (9)	0.058 (3)*	0.25
H15A	0.3970	−0.2793	0.4748	0.087*	0.25
H15B	0.3290	−0.1848	0.4328	0.087*	0.25
H15C	0.2548	−0.2842	0.3990	0.087*	0.25
O2A	0.333 (2)	−0.2650 (10)	0.4138 (7)	0.075 (3)*	0.25
H2AA	0.3521	−0.2536	0.3700	0.113*	0.25
C14A	0.059 (3)	−0.2529 (15)	0.4304 (7)	0.070 (4)*	0.25
H14C	−0.0254	−0.2859	0.3909	0.084*	0.25
H14D	0.0526	−0.1870	0.4158	0.084*	0.25
C15A	0.012 (3)	−0.2491 (14)	0.5119 (7)	0.064 (4)*	0.25
H15G	−0.1703	−0.2413	0.5218	0.096*	0.25
H15D	0.0720	−0.3078	0.5333	0.096*	0.25
H15E	0.1065	−0.1963	0.5324	0.096*	0.25

### Atomic displacement parameters (Å^2^)

**Table:**

	*U*^11^	*U*^22^	*U*^33^	*U*^12^	*U*^13^	*U*^23^
O1	0.0388 (9)	0.0271 (7)	0.0402 (9)	−0.0006 (7)	0.0127 (8)	−0.0014 (6)
N1	0.0301 (9)	0.0343 (9)	0.0266 (8)	0.0049 (7)	0.0037 (7)	−0.0008 (7)
C1	0.0271 (9)	0.0298 (9)	0.0211 (8)	−0.0024 (8)	0.0010 (7)	0.0000 (7)
C2	0.0262 (8)	0.0299 (9)	0.0210 (8)	−0.0017 (8)	0.0025 (7)	−0.0004 (7)
C3	0.0232 (8)	0.0309 (9)	0.0207 (8)	−0.0009 (7)	−0.0029 (7)	−0.0030 (7)
C4	0.0300 (10)	0.0293 (9)	0.0295 (10)	0.0002 (8)	0.0042 (8)	0.0015 (8)
C5	0.0260 (9)	0.0317 (10)	0.0275 (9)	−0.0011 (8)	0.0057 (8)	−0.0003 (8)
C6	0.0227 (8)	0.0274 (9)	0.0194 (8)	−0.0032 (7)	−0.0006 (6)	−0.0045 (6)
C7	0.0228 (9)	0.0271 (8)	0.0234 (9)	−0.0036 (7)	0.0010 (7)	−0.0033 (7)
C8	0.0233 (9)	0.0317 (9)	0.0217 (8)	−0.0027 (7)	0.0019 (7)	−0.0016 (7)
C9	0.0221 (8)	0.0294 (9)	0.0237 (9)	−0.0041 (7)	−0.0008 (7)	−0.0007 (7)
C10	0.0209 (8)	0.0298 (8)	0.0216 (8)	−0.0026 (7)	−0.0017 (7)	−0.0006 (7)
S1	0.0333 (4)	0.0323 (4)	0.0279 (3)	0.0023 (3)	0.0062 (3)	0.0037 (3)
C11	0.0314 (11)	0.0398 (12)	0.020 (2)	−0.0038 (9)	0.0043 (14)	0.0031 (14)
C12	0.0222 (15)	0.0416 (14)	0.0243 (13)	−0.0012 (10)	0.0021 (10)	0.0018 (10)
C13	0.0248 (16)	0.033 (2)	0.0298 (17)	0.0007 (17)	−0.0037 (12)	−0.0028 (19)
S1A	0.0227 (19)	0.031 (3)	0.023 (2)	−0.003 (2)	0.0025 (15)	0.003 (2)
C11A	0.0222 (15)	0.0416 (14)	0.0243 (13)	−0.0012 (10)	0.0021 (10)	0.0018 (10)
C12A	0.0314 (11)	0.0398 (12)	0.020 (2)	−0.0038 (9)	0.0043 (14)	0.0031 (14)
C13A	0.0248 (16)	0.033 (2)	0.0298 (17)	0.0007 (17)	−0.0037 (12)	−0.0028 (19)

### Geometric parameters (Å, °)

**Table:**

O1—C7	1.250 (3)	C11—H11	0.93
N1—C3	1.360 (3)	C12—C13	1.418 (8)
N1—H1A	0.86	C12—H12	0.93
N1—H1B	0.86	C13—H13	0.93
C1—C2	1.382 (3)	S1A—C11A	1.70 (2)
C1—C6	1.404 (3)	C11A—C12A	1.373 (17)
C1—H1	0.93	C11A—H11A	0.93
C2—C3	1.410 (3)	C12A—C13A	1.40 (2)
C2—H2	0.93	C12A—H12A	0.93
C3—C4	1.407 (3)	C13A—H13A	0.93
C4—C5	1.384 (3)	O2—C14	1.416 (9)
C4—H4	0.93	O2—H2B	0.82
C5—C6	1.402 (3)	C14—C15	1.504 (10)
C5—H5	0.93	C14—H14A	0.97
C6—C7	1.475 (3)	C14—H14B	0.97
C7—C8	1.472 (3)	C15—H15A	0.96
C8—C9	1.346 (3)	C15—H15B	0.96
C8—H8	0.93	C15—H15C	0.96
C9—C10	1.446 (3)	O2A—C14A	1.449 (9)
C9—H9	0.93	O2A—H2AA	0.82
C10—C13	1.355 (6)	C14A—C15A	1.509 (9)
C10—C13A	1.45 (2)	C14A—H14C	0.96
C10—S1A	1.701 (8)	C14A—H14D	0.96
C10—S1	1.721 (2)	C15A—H15G	0.96
S1—C11	1.719 (5)	C15A—H15D	0.96
C11—C12	1.377 (5)	C15A—H15E	0.96
			
C3—N1—H1A	120.0	C11—C12—H12	124.2
C3—N1—H1B	120.0	C13—C12—H12	125.2
H1A—N1—H1B	120.0	C10—C13—C12	114.8 (5)
C2—C1—C6	121.7 (2)	C10—C13—H13	123.3
C2—C1—H1	119.1	C12—C13—H13	121.9
C6—C1—H1	119.1	C11A—S1A—C10	93.1 (12)
C1—C2—C3	120.41 (19)	C12A—C11A—S1A	111 (2)
C1—C2—H2	119.8	C12A—C11A—H11A	123.4
C3—C2—H2	119.8	S1A—C11A—H11A	125.1
N1—C3—C4	121.1 (2)	C11A—C12A—H12A	126.4
N1—C3—C2	120.8 (2)	C13A—C12A—H12A	118.7
C4—C3—C2	118.0 (2)	C12A—C13A—C10	109.7 (16)
C5—C4—C3	121.0 (2)	C12A—C13A—H13A	127.1
C5—C4—H4	119.5	C10—C13A—H13A	122.4
C3—C4—H4	119.5	C14—O2—H2B	111.5
C4—C5—C6	121.2 (2)	O2—C14—C15	114.9 (10)
C4—C5—H5	119.4	O2—C14—H14A	106.6
C6—C5—H5	119.4	C15—C14—H14A	108.0
C5—C6—C1	117.7 (2)	O2—C14—H14B	109.2
C5—C6—C7	123.89 (19)	C15—C14—H14B	110.1
C1—C6—C7	118.42 (19)	H14A—C14—H14B	107.9
O1—C7—C8	120.21 (19)	C14—C15—H15A	110.2
O1—C7—C6	120.36 (19)	C14—C15—H15B	108.2
C8—C7—C6	119.43 (18)	H15A—C15—H15B	109.5
C9—C8—C7	121.9 (2)	C14—C15—H15C	109.9
C9—C8—H8	119.1	H15A—C15—H15C	109.5
C7—C8—H8	119.1	H15B—C15—H15C	109.5
C8—C9—C10	125.0 (2)	C14A—O2A—H2AA	107.3
C8—C9—H9	117.5	O2A—C14A—C15A	111.5 (9)
C10—C9—H9	117.5	O2A—C14A—H14C	103.1
C13—C10—C9	127.7 (3)	C15A—C14A—H14C	133.2
C13A—C10—C9	128.8 (8)	O2A—C14A—H14D	95.1
C13A—C10—S1A	111.0 (8)	C15A—C14A—H14D	103.6
C9—C10—S1A	119.9 (3)	H14C—C14A—H14D	103.7
C13—C10—S1	110.6 (3)	C14A—C15A—H15G	110.4
C9—C10—S1	121.65 (17)	C14A—C15A—H15D	108.7
C11—S1—C10	91.9 (2)	H15G—C15A—H15D	109.5
C12—C11—S1	112.1 (4)	C14A—C15A—H15E	109.4
C12—C11—H11	124.0	H15G—C15A—H15E	109.5
S1—C11—H11	123.9	H15D—C15A—H15E	109.5
C11—C12—C13	110.6 (5)		
			
C6—C1—C2—C3	0.2 (3)	C13A—C10—S1—C11	42 (5)
C1—C2—C3—N1	−177.0 (2)	C9—C10—S1—C11	178.1 (3)
C1—C2—C3—C4	0.9 (3)	S1A—C10—S1—C11	−4.5 (4)
N1—C3—C4—C5	176.8 (2)	C10—S1—C11—C12	0.6 (3)
C2—C3—C4—C5	−1.0 (3)	S1—C11—C12—C13	−0.7 (5)
C3—C4—C5—C6	0.0 (4)	C13A—C10—C13—C12	−7.3 (10)
C4—C5—C6—C1	1.0 (3)	C9—C10—C13—C12	−178.3 (4)
C4—C5—C6—C7	179.4 (2)	S1A—C10—C13—C12	154 (4)
C2—C1—C6—C5	−1.1 (3)	S1—C10—C13—C12	−0.2 (6)
C2—C1—C6—C7	−179.63 (19)	C11—C12—C13—C10	0.6 (7)
C5—C6—C7—O1	176.5 (2)	C13—C10—S1A—C11A	−20 (4)
C1—C6—C7—O1	−5.2 (3)	C13A—C10—S1A—C11A	−0.8 (17)
C5—C6—C7—C8	−3.9 (3)	C9—C10—S1A—C11A	−175.2 (14)
C1—C6—C7—C8	174.47 (19)	S1—C10—S1A—C11A	7.3 (14)
O1—C7—C8—C9	−2.4 (3)	C10—S1A—C11A—C12A	0.0 (13)
C6—C7—C8—C9	178.02 (19)	S1A—C11A—C12A—C13A	0.7 (16)
C7—C8—C9—C10	−179.1 (2)	C11A—C12A—C13A—C10	−1(2)
C8—C9—C10—C13	180.0 (4)	C13—C10—C13A—C12A	4(2)
C8—C9—C10—C13A	11.2 (11)	C9—C10—C13A—C12A	175.1 (18)
C8—C9—C10—S1A	−175.4 (4)	S1A—C10—C13A—C12A	1(2)
C8—C9—C10—S1	2.0 (3)	S1—C10—C13A—C12A	−136 (6)
C13—C10—S1—C11	−0.2 (4)		

### Hydrogen-bond geometry (Å, °)

**Table:**

*D*—H···*A*	*D*—H	H···*A*	*D*···*A*	*D*—H···*A*
N1—H1A···O1^i^	0.86	2.16	2.931 (3)	149
N1—H1B···Cg1^ii^	0.86	2.80	3.597 (3)	156

Symmetry codes: (i) −*x*, *y*+1/2, −*z*+1/2; (ii) −*x*+1/2, −*y*, *z*+1/2.
